# Prevalence and predictors associated with modern contraceptive method utilization among women in the nomadic community of Ethiopia: a cross-sectional study

**DOI:** 10.1186/s40834-024-00272-0

**Published:** 2024-04-02

**Authors:** Gebru Gebremeskel Gebrerufael, Bsrat Tesfay Hagos

**Affiliations:** 1 Department of Statistics, College of Natural and Computational Science, Adigrat University, Adigrat, P.O. Box 50, Ethiopia; 2 Department of Statistics, College of Natural and Computational Science, Mekelle University, Mekelle, P.O. Box 231, Ethiopia

**Keywords:** Ethiopia, Logistic regression, Nomadic community, Modern contraceptive method

## Abstract

**Background:**

Ethiopia is one of the countries in sub-Saharan Africa with the lowest prevalence of the use of modern contraceptive methods. On the frequency and determinants of modern contraceptive method in the Ethiopian women who live a nomadic lifestyle, there is, however, scant research. Therefore, the purpose of this study was to evaluate the factors that influence how often women in Ethiopia’s nomadic tribes use modern contraceptive method.

**Methods:**

In the nomadic community of Ethiopia, a community-based retrospective cross-sectional study was carried out between January 18 and June 27, 2016. From the 15,683 nationally representative datasets on the 2016 Ethiopian Demography and Health Survey, a sample of 3,415 women from nomadic communities was chosen. To determine factors linked to modern contraceptive method usage within the nomadic group, a multivariable logistic regression model analysis was considered.

**Result:**

In the nomadic population of Ethiopia, 10% (95% CI (9.10, 11.1)) of respondents reported using modern contraceptive method overall. The most popular way to use modern contraceptive method was through injection (73.5%). In the multivariable logistic regression model analysis, secondary and above-educated husbands (AOR = 1.6, 95% CI (1.01, 2.24)) and primarily educated husbands (AOR = 1.4, 95% CI (1.027, 2.0)), rich (AOR = 1.6, 95% CI (1.03, 2.74)) and middle wealth index (AOR = 1.58, 95% CI (1.25, 2.38)), public health place of delivery (AOR = 2.5, 95% CI (1.55, 3.0)), being a working husband (AOR = 3.8, 95% CI (1.96, 4.22)), and respondents working (AOR = 1.5, 95% CI (1.04, 1.79)) were positively critical risk predictors associated with modern contraceptive method usages. Compared to the Somali region, women living in the Afar (AOR = 2.58, 95% CI (1.68, 3.95)) and Benshangul-Gumuz (AOR = 3.40, 95% CI (2.22, 5.21)) regions had higher use of modern contraceptive method.

**Conclusion:**

In Ethiopia, modern contraceptive method usage is still quite uncommon among women who live in nomadic communities. Therefore, in order to increase modern contraceptive method service utilization and improve the wealth index of households, two key strategies, government professionals and concerned body service providers should pay special attention to educational opportunities for husbands and give valuable and effective counseling information during child delivery for women.

**Supplementary Information:**

The online version contains supplementary material available at 10.1186/s40834-024-00272-0.

## Introduction

Use of modern contraceptive methods (MCMs) is one of the family planning (FP) programs’ most helpful functions. It may enhance people’s freedom to choose their family size in their household. This is one of the most comprehensive approaches to reduce the maternal mortality rate [1, 2]. The World Health Organization’s World Statistics Report states that between 1990 and 2015, MMR trends declined overall, in South Asia (538 to 176), in SSA (987 to 546), and in Ethiopia (1250 to 353).

The total MMR in low-income nations is 239, which is about 20 times greater than in high-income nations [3]. Since 1980, the Ethiopian Ministry of Health has implemented a variety of programs to reduce MMR and sickness, including as increasing the use of contraceptive services in the country’s medical institutions. According to official United Nations population projections and estimations, the world’s population will be somewhere between 7.3 billion and 10.7 billion people in 2050. Low-income nations account for 96% of the global population increase annually [4, 5].

Globally, 645 million women have their needs met by FP, however around 222 million do not have access to FP. Nine children under the age of five die in Africa per year, or 4.8 million children [3, 6].

With the highest MMR, population growth rate, total fertility rate, largest unmet need for FP, and lowest MCM usage and prevalence rates worldwide, SSA nations have faced some of the most critical population and reproductive health issues [4, 7, 8].

With a population of 112 million and a total fertility rate of 4.6 children per woman (2.3 in urban areas and 5.2 in rural areas), Ethiopia is one of the SSA countries with the highest population. Only less than 23% of women in the reproductive age group are currently using MCM, which is still very low to affect fertility when compared to Nigeria [7, 9–11].

Current Ethiopian women have gradually increased their use of modern contraceptive methods over the past 15 years, going from 6 to 38% of women using MCM between 2000 and 2018 [9, 10, 12]. The largest increase has been in the use of injectables, which increased from 3 to 23% of women between 2000 and 2016, followed by growth in the use of implants, which increased from 1 to 8% of women between 2000 and 2016 [9]. Moreover, the regions experience high MMR due to general poor health conditions, high incidence of unintended pregnancies, insufficient access to medical care, unplanned deliveries, and unsafe abortions [3, 13, 14].

Additionally, because Ethiopian couples find it challenging to provide enough food and health care for all of their children, a high fertility rate carries the crucial risk factor of a high child mortality rate. Children may therefore be more susceptible to infections and severe malnutrition [7]. The usage of MCM by the population varies significantly by area in Ethiopia. That is, compared to the national figure of 35% reported on the 2016 EDHS, MCM utilization is still relatively low in several parts of Ethiopia, especially in the nomadic living community of Ethiopia (i.e., reported to be in Somali (1%), Afar (12%), and Benishangul-Gumuz (28%)) [9].

Household wealth index, husband education, religion, and women’s working status were among the predictors linked to MCM, according to evidence from various literatures [2, 7, 15–17].

Ethiopia is working to improve the coverage, quality, and use of skilled care, community health initiatives, and other efforts made by government and non-governmental organizations in order to increase the rate of MCM utilization, but it is also raising awareness about how to use it and its benefits. In the nations of Afar, Somalia, and Benishangul-Gumuz, it is still incredibly low [8, 9]. This demonstrates that there have been few research conducted in the study area that examine the percentage of women who use MCM and its related risk factors. In order to implement suitable and specialized interventions, it is also crucial to determine the prevalence and major risk variables in the real local situation. Therefore, this study was aimed at assessing predictors associated with MCM utilization among women in the nomadic community of Ethiopia.

## Methods and materials

### Study design, period, and setting

For this analysis, a community-based retrospective cross-sectional study using secondary analysis of the 2016 EDHS data set was carried out in the nomadic community of Ethiopia from January 18 to June 27, 2016 [9]. Afar, Benishangul-Gumuz, and Somali are the three regional states in Ethiopia where nomadic life communities may be found [9]. The Central Statistical Agency (CSA), the Ethiopian Minister of Health (EMOH), and the Ethiopian Public Health Institute (EPHI) conducted the fourth recent EDHS survey, which was reported in 2016 [9].

### Study population, data source, and sampling procedure

**Fig. 1:**
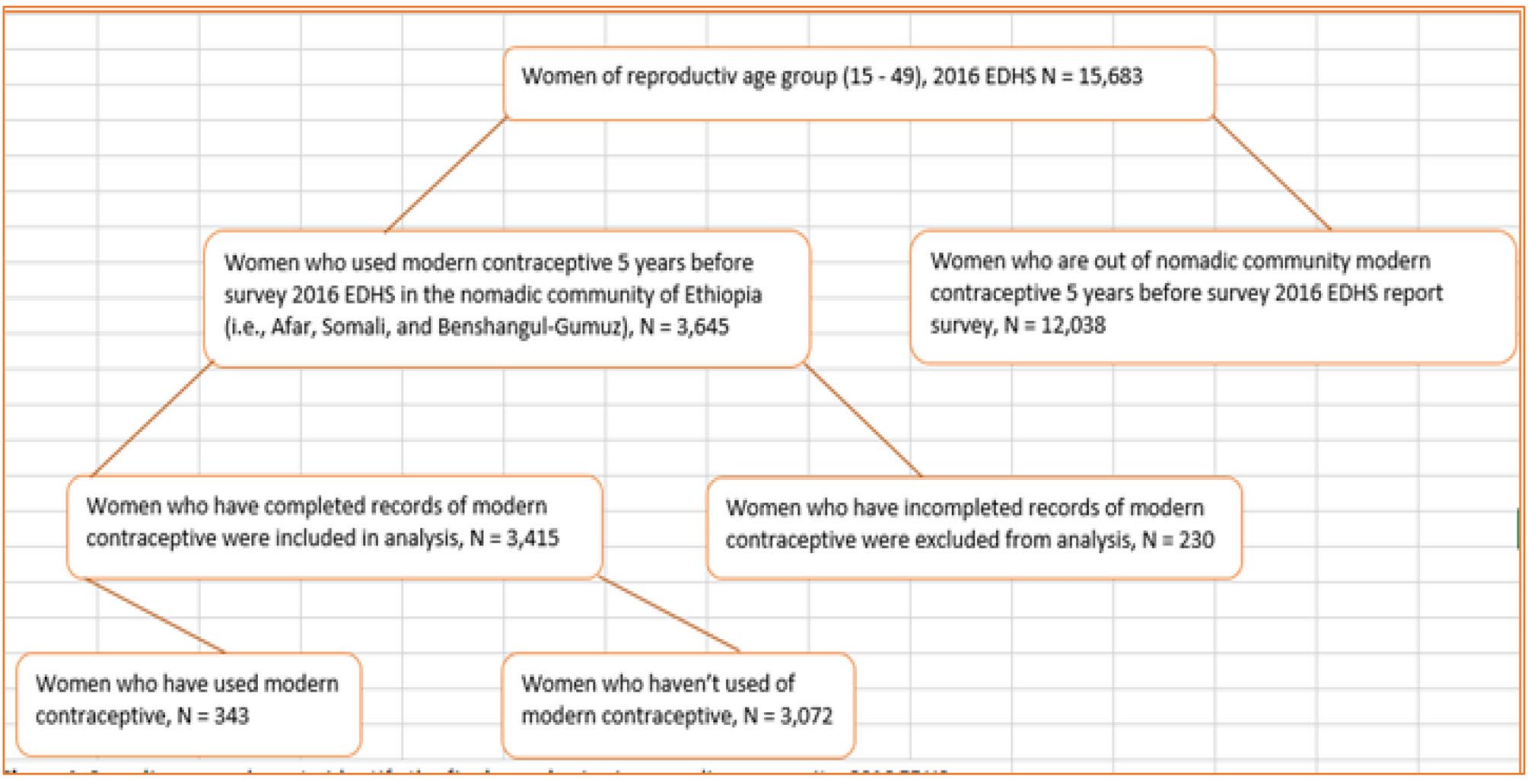
Sampling procedures to identify the final sample selected numbers in nomadic community, 2016 EDHS

The study covered all female participants who were between the 15–49 age ranges. The Ethiopian Demography and Health Survey (EDHS) from 2016 provided the data set for this investigation. A two-stage stratified sampling technique was utilized for the 2016 EDHS. 645 strata of Enumeration Areas (EAs) were chosen in the first round, 443 of which were in rural areas and 202 in urban areas. The EA was used as a census counting unit because it is a geographic area with a sufficient number of households inside it. 28 families per EA were chosen in the second stage, with each EA having an equal chance of being chosen. The 2016 Full EDHS Report included a detailed explanation of the sampling selection process [9]. Since these areas had the lowest prevalence rates of using modern contraceptives, according to the 2016 EDHS report, Afar, Somalia, and Benishangul-Gumuz regional states were chosen. The final analysis included a sample of 3,415 women who provided information within the five years prior to the survey [**see** Fig. 1].

### Variables of the study

#### Response variable

The response variable of this study was modern contraceptive method (MCM) utilization among nomadic community women in Ethiopia. It is a categorical variable (yes or no). Thus, the response variable for the i^th^ woman is dichotomous, represented by a random variable Yi that is coded as the value “1” with probability of success (used MCM) and the value “0” with probability of failure (not used MCM), such that

$$ {Y_i} = \left\{ {\begin{array}{*{20}{c}}{{\rm{1}}\,\,\,\,\,{\rm{if}}\,{\rm{there}}\,{\rm{is}}\,{\rm{used}}\,{\rm{modern}}\,{\rm{contraceptive}}\left( {{\rm{yes}}} \right)}\\{{\rm{0}}\,\,\,\,\,{\rm{if}}\,{\rm{there}}\,{\rm{is}}\,{\rm{not}}\,{\rm{used}}\,{\rm{modern}}\,{\rm{contraceptive}}\left( {{\rm{no}}} \right)}\end{array}} \right.$$

The pill, intrauterine device (IUD), injections, the diaphragm, sterilization, male or female condoms, implants, and lactation amenorrhea contraceptive techniques were the MCMs covered by this study.

#### Independent variables

The independent variables were selected by review of previous literature [2, 3, 7, 11, 18–20]. The independent variables included in the study are given in Table 1.

**Table 1:** Operational definitions and categorizations of independent variables

№	Variables	Categorizations of independent variables
1	Residence	Place of residence (rural, urban)
2	Region	Region (Afar, Somali, Benshangul-Gumuz)
3	Wealth index	Wealth index of household (rich, middle, poor )
4	Religion	Religion (Muslim, Orthodox, others )
5	Sex of household head	Sex of household head (female, male )
6	Age of respondent at 1st birth	Age of respondent at 1st birth (18 and above, 17 and below)
7	Births in last three years	Births in last three years ( no birth, one birth, 2 and above)
8	Place of delivery	Place of delivery (home, public health)
9	Husband education	Husband education level (no education, primary, secondary and above )
10	Husbands occupation	Husbands occupation status (no, yes )
11	Respondent working	Respondent working status (no, yes)

### Operational definitions

In this study, an occupation of nomadic communities of Ethiopia is quite different from urban areas. Such communities rely heavily on traditional livelihood practices that are intimately tied to their nomadic lifestyle. Such occupation in nomadic Ethiopia is intertwined with their connection to nature, livestock, and the communal way of life. The possible reason behind this could be, the characteristics of nomadic women those have a high mobility, depend on traditional knowledge and culture, and also they depends on reliance on natural resources.

### Data management and analysis

The SPSS statistical software application, version 26, was used to enter, code, clean the data before analysis, and then analyze the results. The survey respondents were described and the prevalence rate of MCM was calculated using descriptive statistics, such as frequencies, percentages, and bar charts. Binary logistic regression models that included two or more variables were used to examine associations between independent and response variables. The Hosmer and Lemeshow test and the Wald test were applied to the goodness-of-fit test model. The variance inflation factor (VIF) statistic value was used to determine the degree of multi-collinearity and test for correlation between independent variables. The AOR and 95% CI were used to calculate the statistical connection between the various independent variables and the dependent variables, and *P*-values < 0.05 were regarded as statistically significant.

## Result

### Socio-demographic characteristics of the respondents

A total sample of 3,415 nomadic community women was included in the analysis. Regarding the births in the last three years, it was realized that about 54.4% of those had no child in the births in the last three years. The majority of the participants (79.9%) were Muslims, followed by Orthodox Christians (12.7%). The majority (74.1%) of women was rural residents, and 65.3% were male household heads. More than a two third (66.6%) of the study population were poor, 9.0% were rich, and; a fourth of the study sample (24.3%) were in the middle wealth index of households (24.3%). About the educational level, 74.3% and 10.7% of women of their husbands were not educated, and secondary and above education levels, respectively. The majority of the women did not have an occupation (70.9%). The age at first birth of women 18 and older was 68.5% (see Table 2).

**Table 2:** Socio-demographic characteristics of women in the nomadic community of Ethiopia on January 18 to June 27, 2016 (*n* = 3,415)

Variables	Categories	Frequency	Percentage
Residence	Urban	883	25.9
	Rural	2532	74.1
Region	Afar	1001	29.3
	Benshangual-Gumuaz	1104	32.3
	Somali	1310	38.4
Wealth index	Rich	308	9
	Middle	831	24.3
	Poor	2276	66.6
Religion	Orthodox	434	12.7
	Others	251	7.3
	Muslim	2730	79.9
Sex of household head	Male	2231	65.3
	Female	1184	34.7
Age of respondent at 1st birth	17 and below	1076	31.5
	18 and above	2339	68.5
Births in last three years	2 and above	298	8.7
	1 birth	1260	36.9
	No birth	1857	54.4
Place of delivery	Public facility	450	13.2
	Home	2965	86.8
Husband education	Secondary and above	365	10.7
	Primary	514	15.1
	No education	2536	74.3
Husbands occupation	Yes	1927	56.4
	No	1488	43.6
Respondent working	Yes	995	29.1
	No	2420	70.9

### Prevalence of modern contraceptive methods among nomadic women in Ethiopia

**Fig. 2:**
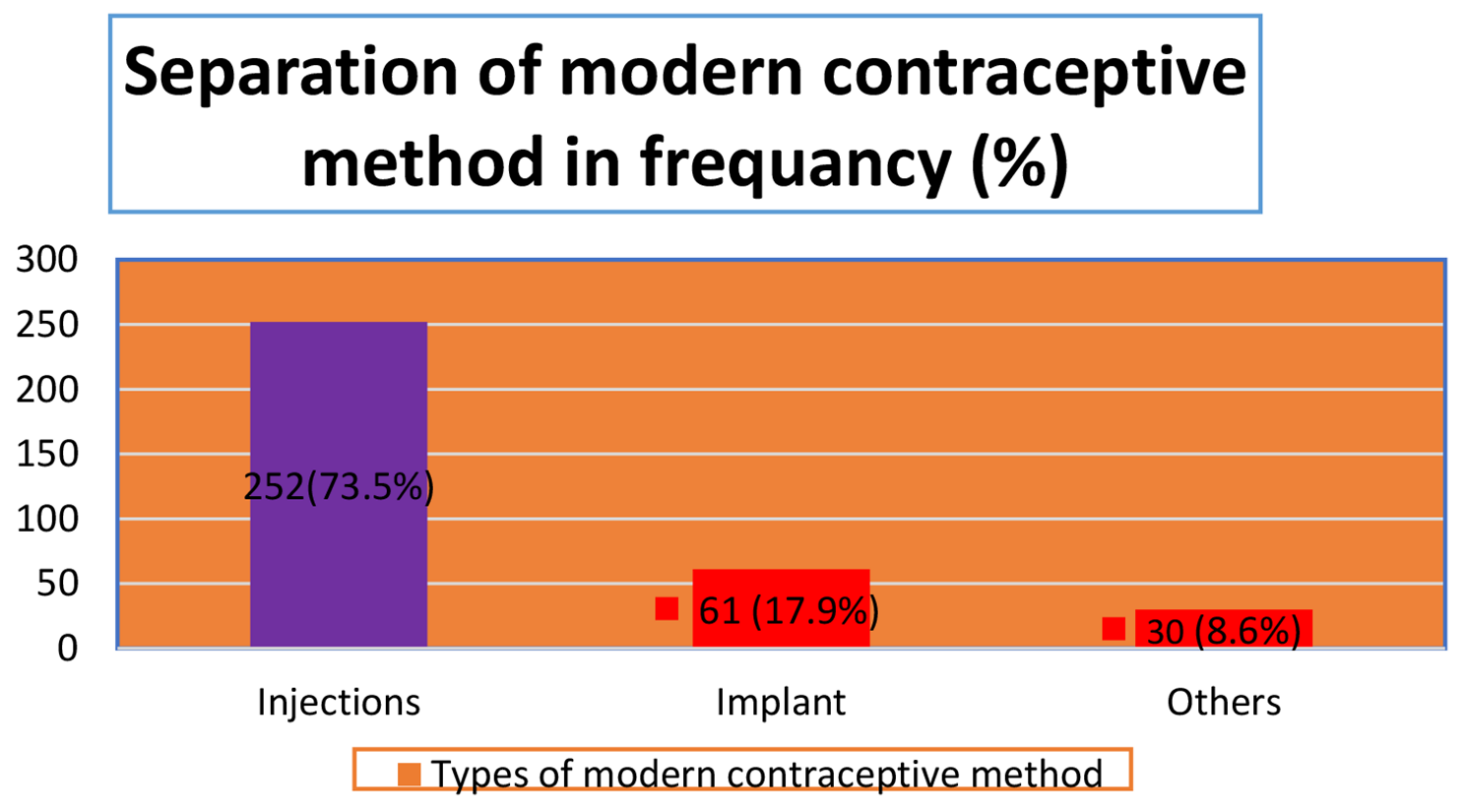
Frequency of utilization on MCM by type in nomadic community women of Ethiopia **N.B**: Others: IUD, pill, female sterilization, standard day method, and lactation amenorrhea.

From a total sample selected of 3,415 nomadic community women in Ethiopia, the overall prevalence of MCM utilization was found to be 343 (10% (95% CI (9.10, 11.1)), and the rest of 3072 (90.0%) did not utilize any MCM. Among 343 MCM utilizers, 252 (75.3%) used injections, 61 (17.9%) used implants, and the remaining 30 (8.6%) of women used other MCM (i.e., pill, IUD, female sterilization, standard day method, and lactation amenorrhea method) (see Tables 2**and** Fig. 2).

### Binary logistic regression and assessment of goodness of fit of the model

Each covariate’s impact on MCM usage was investigated using a binary logistic regression model analysis. Using the Hosmer and Lemeshow test and the Wald test, we first examine the general goodness of fit. Accordingly, the Wald test provided a chi-square value of 341.2 with a *p*-value < 0.000, which would imply a good fit for the model. Similarly, the Hosmer and Lemeshow test found that the observed data was better explained by the model (chi-square value = 1208.9 with *p*-values = 0.165). Furthermore, there is no multi-collinearity issue because the correlation value for all predictors is less than 10 (Table S1).

### Predictors associated with modern contraceptive method utilization among women in the nomadic community of Ethiopia

In the multivariable logistic regression analysis, region, wealth index, religion, place of delivery, husband education, husband occupation, and respondent working have critical risk predictors associated with modern contraceptives in the nomadic community of Ethiopia. Additionally, women who had a work were 1.5 (AOR = 1.5, 95% CI (1.04, 1.79)) times more likely to utilize modern contraceptive methods than those who had no job. The likelihood of being willing to use modern contraceptive methods among Orthodox Christian respondents was 3.0 (AOR = 3.0, 95% CI (2.19, 4.32)) times more likely than that for Muslim respondents.

Respondents in the rich wealth index of households were 1.6 (AOR = 1.6, 95% CI (1.03, 2.74)) times more likely to use modern contraceptives than those in the poor wealth index of households. Similarly, women with a middle wealth index of households were 1.58 (AOR = 1.58, 95% CI (1.25, 2.38)) times more likely to use modern contraceptives than women with a poor wealth index of households. Women whose husbands joined primary education were 1.4 (AOR = 1.4, 95% CI (1.027, 2.0)) times more likely to use modern contraceptive methods than those who did not join any formal education.

Correspondingly, women whose husbands had a secondary and above education level were 1.6 (AOR = 1.6, 95% CI (1.01, 2.24)) times more likely to use modern contraceptives than women whose husbands had a no education level. Besides, women whose husbands had a job were 3.8 (AOR = 3.8, 95% CI (1.96, 4.22)) times more likely to use modern contraceptive methods than those who had not worked.

It was discovered that MCM was critically correlated with the place of delivery. Women who were born in a public health place of delivery were 2.5 times (AOR = 2.5, 95%CI (1.55, 3.0)) more likely to utilize contraceptives than their counterparts.

Finally, women living in the Benshangul-Gumuz were 3.40 (AOR = 3.40, 95% CI (2.22, 5.21)) and Afar region were 2.58 (AOR = 2.58, 95% CI (1.68, 3.95)) more likely to utilize modern contraceptive methods as compared to women living in the Somali region (see Table 3).

**Table 3:** Bivariable and multivariable logistic regression results for predictors associated with MCM utilization among women in nomadic community of Ethiopia, 2016 EDHS (*n* = 3,415)

		Modern contraceptive		Odds Ratio (95% CI)	
**№**	**Variables**	**No**	**Yes**	**COR**	**AOR**
1	Residence (reff.= rural)	2317	215		
	Urban	755	128	1.83 (1.45,2.31)*	1.2 (0.93, 1.56)
2	Region (reff.= Somali)	1273	37		
	Afar	919	82	3.07 (2.10,4.57)*	2.58 (1.68, 3.95)*
	Benshangul–Gumuz	880	224	8.76 (6.12,12.53)*	3.40 (2.22, 5.21)*
3	Wealth index (reff.= poor )	2109	167		
	Rich	253	55	2.75 (1.97,3.82)*	1.6 (1.03, 2.74)*
	Middle	710	121	2.15 (1.68,2.76)*	1.58 ( 1.25, 2.38)*
4	Religion (reff.= Muslim )	2559	171		
	Orthodox	310	124	5.99 (4.62,7.76)*	3.0 (2.19, 4.32)*
	Others	203	48	3.54 (2.49,5.02)*	1.6 (1.01, 2.38)*
5	Sex of household head (reff.=female )	1106	78		
	Male	1966	265	1.91 (1.47,2.49)*	1.13 (0.83, 1.54)
6	Age of respondent at 1st birth (reff.= 18 and above)	2137	202		
	17 and below	935	141	1.60 (1.27,2.01)*	1.07 (0.8, 1.41)
7	Births in last three years (reff.= no birth)	1691	166		
	2 and above	280	18	0.66 (0.39,1.08)	0.63 (0.36, 1.1)
	One birth	1101	159	1.47 (1.17,1.85)*	0.91 (0.69, 1.23)
8	Place of delivery (reff.= home)	2725	240		
	Public health	347	103	3.37 (2.61,4.36)*	2.5 (1.55, 3.0)*
9	Husband education (reff.= no education)	2378	158		
	Secondary and above	286	79	4.16 (3.1,5.59)*	1.6 (1.01, 2.24)*
	Primary	408	106	3.91 (2.99,5.11)*	1.4 (1.027, 2.0)*
10	Husbands occupation (reff.= no )	1438	50		
	Yes	1634	293	5.16 (3.79,7.02)*	3.8 (1.96, 4.22)*
11	Respondent working (reff.= no)	2244	176		
	Yes	828	167	2.57 (2.05,3.22)*	1.5 (1.04, 1.79)*

**N.B**: reff. = reference for the category variables; * Significant at 5% level of significance

Hosmer and Lemeshow Test: Chi-square (1162) = 1208.9 with *p*-value = 0.165

Wald Test: Chi-square (16) = 341.2 with *p*-value = 0.000*

## Discussion

The purpose of this study was to determine the prevalence of MCM use among women in the Ethiopian nomadic community and the variables linked with it. The prevalence of MCM use overall in this retrospective analysis was 10% (95% CI ((9.10, 11.1)). This findings show that these reproductive women used MCM, which is a smaller percentage than the 35% reported from the 2016 EDHS [9].

Similarly, this result was lower than in the Tigray region, Ethiopia, 35.6% [8], Ghana, 33.2% [21], South Africa, 41.8% [21], Edaga-hamus Town, Eastern Zone, Tigray, Ethiopia, 58.5% [3], Benin City, Nigeria, 64.5% [22], Holeta Town, 73% [11], and Mbarara, Uganda, 85% [23]. This variation may be due to socio-demographic features and cultural variation. Moreover, there is a discrepancy in the study time gap between the studies. However, the prevalence of MCM utilization in this study was higher than in the UN Millennium Development Goal (MDG) 2015 report for South Sudan, at 6.8% [4]. This difference might be because of the socio-demographic, cultural, residence, or religious beliefs of the participant. One of the barriers was the longer distance to the public health facilities in rural areas, and there were also more job opportunities for urban residents. Additionally, women who deliver their children in urban areas have better access to public health institutions for various service providers like FP [18].

Women who use MCM make up 73.5% of those who use the injection method, 17.9% of those who use implants, and 8.6% of those who use other advanced methods. It is made up of research conducted in Ethiopia [3, 7, 11, 19, 20]. These findings show that short-acting hormonal MCM, like injections, are preferred by women. IUDs and implants are long-acting reversible methods of pregnancy prevention, although permanent options are more successful [24]. This could be explained by the fact that individuals with higher levels of education are more aware of and have easier access to public health delivery services during childbirth, such as FP.

The use of MCM was also favorably correlated with husband education. The findings of investigations [2, 15, 16, 25, 26] support this conclusion. This may be due to the fact that educated husbands may communicate with their wives more effectively and may be more open to discussing MCM use.

Another element that was positively associated with MCM utilization was the household wealth index. In comparison to impoverished women in homes, rich women were more likely to use MCM. Women in homes with greater socioeconomic status are more likely to use MCM, according to earlier studies [2, 15, 27, 28]. This might be mostly due to the fact that women in wealthy households have greater access to media, are more educated, are able to make their own decisions, and have received superior health care services. The poorest women, on the other hand, could be hesitant to use their medical facilities because they might feel hopeless because they are still scrambling to meet their fundamental needs.

The use of MCM has also been linked to women’s working status. Women who had jobs had a higher likelihood of using MCM than those who did not. This study and others like it have been done [2, 26, 29, 30]. Women who have held multiple jobs are more willing to share their knowledge and expertise about MCM with their coworkers than women who have not held any jobs.

The results of this study demonstrated that one of the important factors linked to MCM was religion. Muslim women were less likely to use MCM than Orthodox Christian, other (Catholic, Protestant, and traditional), and other women. This result is in line with studies that have been done [16, 17, 28]. This may be due to the widespread acceptance by the Muslim populace of a sacred book that forbids FP [17]. Additionally, further research is required to fully understand this result’s feature.

Finally, there have been many variations in how MCM has been used across the world. Compared to the Somali region (used as a reference region), women in the Afar and Benshangul-Gumuz regions used MCM more frequently. This outcome was in line with research from Malawi [26] and Ethiopia [2]. This regional variance may be explained by the fact that different regions implement MCM differently, including in terms of FP service providers. The highest rate of under-five child mortality in Ethiopia is caused by MCM’s inaccessibility [30, 31]. This suggests that the availability of MCM will increase health complexity for moms while lowering the death rate for children under the age of five.

This study demonstrated a positive correlation between MCM use and the place of delivery.

Women who were born in a public health place of delivery were 2.5 times (AOR = 2.5, 95% CI (1.55, 3.0)) more likely to use modern contraceptives than their counterparts. The finding is consistent with studies conducted in Ethiopia [32, 33]. This may be explained by the fact that women who had easier access to childbirth care in a public hospital setting may be more familiar with other maternal health services. It may also have given medical staff the chance to counsel patients on the importance of modern contraceptive services.

## Conclusion

The prevalence of MCM utilization in the nomadic community of Ethiopia was 10%, which was still quite low (and much lower than the national average). The most popular MCM method used was injection (73.5%). Educated husbands, respondent working women, working husbands, public health place of delivery, Orthodox Christian women, rich and middle wealth index, and regions were predictors of positively associated MCM with utilization.

Therefore, improving the wealth index of households and providing educational opportunities should be given special attention by government officials and related body service providers as important initiatives to boost the usage of MCM services. To increase the use of modern contraceptives, it is also essential to increase the accessibility and usage of maternal health care services, such as the use of public health delivery facilities.

### Electronic Supplementary Material

Below is the link to the electronic supplementary material.

**Additional file: Table S1:** The VIF value among predictor variables on January 18 to June 27, 2016, in the nomadic community of Ethiopia.

## Data Availability

The survey datasets used in this study were based on a publicly available dataset that is freely available online with no participant’s identity from http://www.dhsprogram.com/data/available-datasets.cfm.

## Abbreviations

AOR: Adjusted Odds Ratio
CI: Confidence Intervals
COR: Crude Odds Ratio
CSA: Central Statistical Agency
EAs: Enumeration Areas
EDHS: Ethiopian Demographic and Health Survey
EMOH: Ethiopian Ministry of Health
EPHI: Ethiopian Public Health Institute
FP: Family Planning
MCM: Modern Contraceptive Methods
MDG: Millennium Development Goal
MMR: Maternal Mortality Rate
IUD: Intrauterine Device
SPSS: Statistical Package for Social Science
SSA: Sub-Saharan Africa
UN: United Nations
VIF: Variance Inflation Factor
