# Supplementary material for: Prevalence and predictors associated with modern contraceptive method utilization among women in the nomadic community of Ethiopia: a cross-sectional study
Source: Contracept Reprod Med. 2024 Apr 2;9:11. doi: 10.1186/s40834-024-00272-0 (PMC10988917; doi:10.1186/s40834-024-00272-0)
Supplement: Supplementary file 1 — Additional file: Table S1: The VIF value among predictor variables on January 18 to June 27, 2016, in the nomadic community of Ethiopia. [file 40834_2024_272_MOESM1_ESM.docx]

**Supplementary materials**

**Additional file: Table S1:** The VIF value among predictor variables on January 18 to June 27, 2016, in the nomadic community of Ethiopia.

| **№** | **Variables** | **VIF** |
| --- | --- | --- |
| 1 | Residence (reff. = rural) |  |
|  | Urban | 1.37 |
| 2 | Region (reff. = Somali) |  |
|  | Afar | 1.58 |
|  | Benshangul-Gumuz | 2.96 |
| 3 | Wealth index (reff. = poor) |  |
|  | Rich | 1.33 |
|  | Middle | 1.47 |
| 4 | Religion (reff. = Muslim) |  |
|  | Orthodox | 1.69 |
|  | Others | 1.42 |
| 5 | Sex of household head (reff. =female) |  |
|  | Male | 2.52 |
| 6 | Age of respondent at 1^st^ birth (reff. = 18 and above) |  |
|  | 17 and below | 1.59 |
| 7 | Births in last three years (reff. = no birth) |  |
|  | 2 and above | 1.26 |
|  | One birth | 2.04 |
| 8 | Place of delivery (reff. = home) |  |
|  | Public health | 1.38 |
| 9 | Husband education (reff. = no education) |  |
|  | Secondary and above | 1.45 |
|  | Primary | 1.46 |
| 10 | Husband’s occupation (reff. = no) |  |
|  | Yes | 3.18 |
| 11 | Respondent working (reff. = no) |  |
|  | Yes | 1.55 |
|  | Mean VIF | 1.77 |
